# Raman spectra for plastics identification (RaSPI) and Raman maps for plastics identification (RaMPI) datasets

**DOI:** 10.1038/s41597-026-07103-8

**Published:** 2026-03-30

**Authors:** Úna. E. Hogan, H. B. Voss, Benjamin Lei, Avery E. Bec, Xinyi Feng, Rodney D. L. Smith

**Affiliations:** 1 Department of Chemistry, University of Waterloo, 200 University Avenue W., Waterloo, Ontario N2L 3G1 Canada; 2 Waterloo Institute for Nanotechnology, University of Waterloo, 200 University Avenue W., Waterloo, Ontario N2L 3G1 Canada

**Keywords:** Raman spectroscopy, Analytical chemistry, Environmental monitoring

## Abstract

Efforts to expedite accurate identification of environmental plastics pollution have strong focus on machine learning (ML) techniques. We published two Raman spectroscopy datasets to support the development of next-generation ML methods. The Raman spectra for plastics identification (RaSPI) dataset presents 402 high-quality Raman spectra with <1 cm^−1^ resolution between 100 and 4000 cm^−1^. RaSPI spans 14 plastic types and has variability in (unknown) additives. The Raman maps for plastics identification dataset (RaMPI) contains 34 two-dimensional spectroscopic maps containing 33,119 spectra. RaMPI spectra offer <1 cm^−1^ resolution across the fingerprint region, with significant variability in signal:noise ratios that is useful for methodology testing and validation. Both datasets contain data from pristine samples and from environmental pollution. Spectra across both datasets have been manually assigned as one of 14 different plastic classifications. The consistency and quality of these datasets make them high-value resources for researchers active in diverse topics, including training ML models for microplastics research, for developing spectroscopic processing algorithms, or for those seeking datasets to test their methodologies against.

## Background & Summary

From the invention of the first human-made synthetic polymer, a compound called Bakelite formed from the monomers phenol and formaldehyde in 1909^1,2^, to recent advances in high-tech flexible plastics used for wearable electronic devices^3^, plastics have been an integral and prevalent part of modern human life for over 100 years. In 2023, the global production of short-life plastics was predicted to be over 159 Megatons (Mt)^4^, with production estimated to continue to increase with further increases in the global world population^5^. With global plastic recycling rates of only 9%^6^, the majority of this plastic finds its way into landfill and the environment, both of which lead to microplastics pollution. Plastic particles have now been found throughout the natural world, including in deep ocean, atop mountains, and even at Point Nemo – located in the deep the southern Pacific Ocean and believed to be the furthest place on Earth from civilization^7–9^. With such a global prevalence, it is unsurprising that plastic particles have also been found in human food-sources such as fish, meat, vegetables and drinking water^10–13^. These particles have been found to enter the human body, being identified within placenta, lungs, blood and brain matter^14–16^. Current scientific methods to identify and quantify these plastic particles are labor intensive and time consuming, which limits our understanding of the true extent of the problems caused by these pervasive pollutants and delays development of legislation to address it. To better understand the fate and transport of microplastics particles in the environment, the ability to identify these particles quickly and accurately is necessary.

Chemical identification of microplastic particles is an important part of current studies into environmental pollution. Vibrational spectroscopy techniques such as Raman spectroscopy have shown promise as effective techniques for identification of plastic components. The chemical-specificity of Raman spectroscopy combines with Raman-inactivity of water to make the technique particularly useful for microplastics identification in environmental samples. Raman spectroscopy has been applied for plastics identification on plastic^17^ pollution in remote lakes in China^18^, plastic particles in surface and groundwater in Greece^19^ and analysis of plastic fibers in beverages^20^. Conventional strategies to identify plastics from their Raman spectra rely upon statistical comparison of an experimental spectrum with standards compiled within a spectroscopic database/library. Such analyses, however, are inherently limited by their reliance upon spectra acquired from pristine, chemically pure samples.

Real-world plastics are complex materials that contain diverse additives and undergo chemical degradation with time. It is estimated that over 10,000 individual chemical additives are used in the plastics industry, including dyes, plasticizers and stabilisers^21–23^. The dyes and parent plastics are known to undergo degradation through processes such as UV-facilitated photo-oxidation and chemical reaction within impure water sources; they have also been shown to adsorb chemical pollutants such as pesticides^24–29^. The complexity of the initial composites and the ability for individual components to undergo degradation introduce significant sample-to-sample variability. The variability introduced by structural changes are also diverse, including the introduction of fluorescence processes, addition of new vibrational peaks, the masking or complete disappearance of peaks or the shifting of peaks. The extreme complexity introduced by these features often renders human oversight necessary for all statistical comparisons to standards. Whilst Raman spectroscopy is a powerful tool for plastics identification, and traditional library searches continue to be the *de facto* standard, these are only a few of the very clear limitations.

Machine learning (ML) algorithms have shown promise for identifying plastics from their Raman spectra without the need for human oversight. Given sufficient data quality and quantity, these models can identify the critical components of a spectrum and are used to identify plastics even in the presence of unknown additives and degradation. ML models based on Raman spectra have been trained to identify microplastic particles from one of 6 plastic types^30^, to similarly classify plastic particles from 14 different plastic types^31^, to quantify marine litter in coastal environments^32^, and to classify microplastics from a household environment^33^. Judicious selection of data parameters has been shown to be important, with extension of spectroscopic range to higher frequencies improving classification accuracy^34^. Access to high-quality Raman spectra remains limited despite the extensive interest in developing the analytical capabilities using ML. Research teams have independently created custom databases, but this has generated significant variability in data resolution and acquisition parameters. Data limitations extend to include variations in chemically pure samples and commercial plastics, and pristine and environmentally degraded plastics^35–37^. The SLOPP and SLOPP-E spectral databases are well-established sets of spectra that were published in 2020^38^, which provided open-access and easily available information for microplastics identification. These databases contain a blend of pristine and environmentally degraded samples, but also relatively low spectroscopic resolution and inconsistent data spacing and spectral ranges. The *OpenSpecy* database was created to compile publicly available data and facilitate microplastics research^39,40^. Drawing spectra from a diverse range of sources, the database contains variability in data parameters and many spectra that are not related to plastics. Such databases provide excellent advances in data availability for Raman spectra on plastics but also necessitate that researchers invest significant effort into data cleaning to integrate the data into their workflows.

Herein we publish two labeled datasets that will be valuable for researchers in microplastics research and in the development of ML algorithms and models. The *Raman Spectra for Microplastics Identification* (RaSPI) dataset consists of 402 high-resolution Raman spectra measured between 100 and 4000 cm^−1^ on diverse plastic samples. The acquisition of selected plastic types from varied sources results in variations in unknown additives across each plastic type. The high quality of spectra and variability of samples makes the RaSPI dataset useful for exploring ML model development. Manufacturers and commercial products rarely list all additives within plastics. The variation of suppliers therefore introduces mixtures of unknown additives that are expected for real-world samples. The *Raman spectral Maps for Microplastics Identification* (RaMPI) is a collection of 34 2-dimensional Raman microscopic maps of plastic particles consisting of 33,119 individual spectra, 16,519 of which are of plastic particles, with the rest being blank background spectra. This dataset was measured between 700 and 1800 cm^−1^ and provides substantial variations in signal:noise ratio. This variability in data quality makes RaMPI a useful dataset to test methodology. All spectra across these two datasets are individually labeled with the plastic that they contain, which includes blank spectra and 14 different types of plastic. They are published as a support for researchers interested in strengthening our ability to identify and quantify microplastics, but also provide value for researchers developing, training and testing ML models broadly focused on spectroscopic analysis. All spectra in RaSPI are provided in their unprocessed form as well as a post-processed form, which involved baseline subtraction, removal of cosmic rays, interpolation of all spectra to a shared wavenumber grid between 225 and 3950 cm^−1^, and normalization. Each spectrum is labeled with a sample code. Sample codes are each linked to a plastic type, the source of the plastic, the color of the sample, and extra notes. The RaMPI database is shared in several forms, including the original files from the proprietary Renishaw WiRE software (version 5.5, which is readable using open source code^41^), as individual comma-separated value files, and as Python dictionaries exported to a Python pickle file. Portions of these datasets have been previously used to train ML classification models for microplastics identification^31,34^.

## Methods

### Materials

**Fig. 1:**
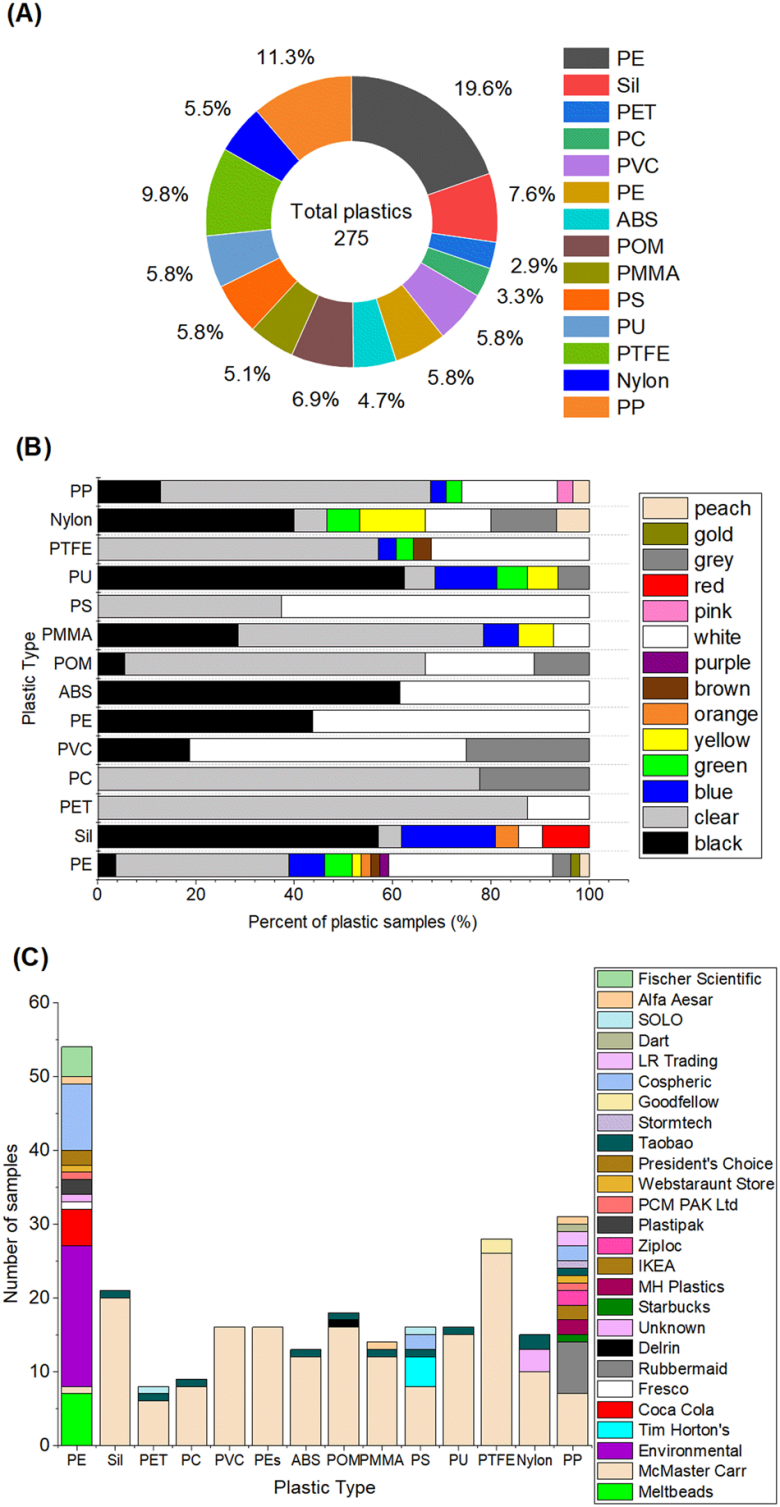
Description of plastic samples contained within the datasets. (**A**) Distribution of each of the 14 types of plastic within the 275 total plastic samples represented within the datasets. (**B**) Colour of plastics as a percentage of total number of plastic samples. (**C**) Source of plastic samples by plastic type.

A total of 275 plastic samples were collected from a variety of sources including commercial suppliers such as Sigma Aldrich, retail stores such as Tim Horton’s and Starbucks, and plastics extracted from environmental pollution. All spectra in the RaSPI dataset are labeled with a unique Spectra ID, the color of the plastic, the origin (source) of the sample, additional notes (if applicable), the laser wavelength used for the spectral acquisition (633 nm or 532 nm) and the type of plastic. The 14 different types of plastic represented within the dataset, and their abbreviations, are: polyester (PEs), polyethylene (PE), polyurethane (PU), polypropylene (PP), poly(methyl methacrylate) (PMMA), polyethylene terephthalate (PET), acrylonitrile butadiene styrene (ABS), polystyrene (PS), polyoxymethylene (POM), polyvinyl chloride (PVC), polycarbonate (PC), silicone (S) and polytetrafluoroethylene (PTFE). The dataset contains a variation of both plastic types, (Fig. 1a), colors (Fig. 1b) and source (Fig. 1c). A total of 402 Raman spectra were taken of these 275 plastics, 226 taken with a 633 nm laser and 176 taken with a 532 nm laser.

**Fig. 2:**
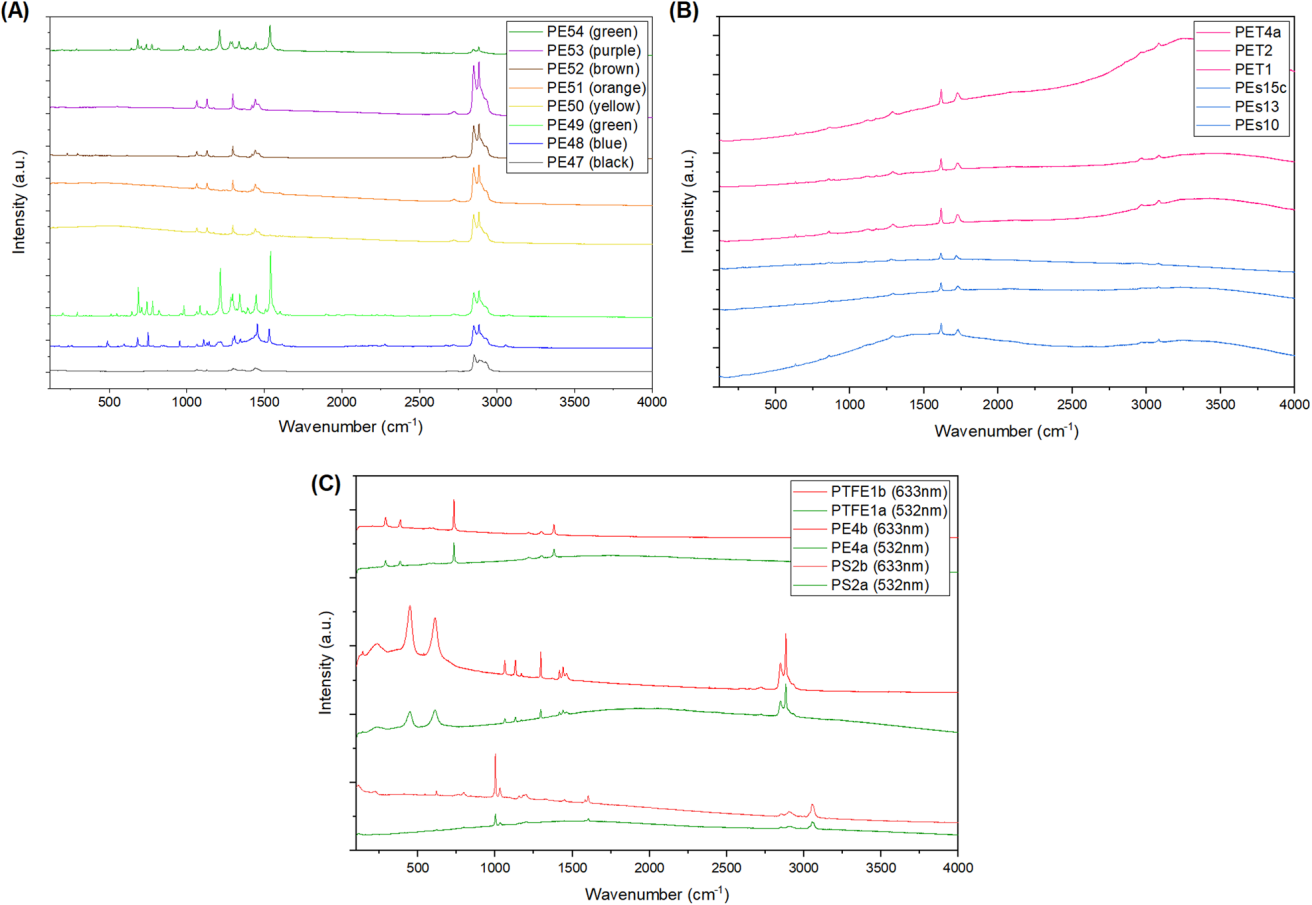
Selected comparisons of individual spectra within the database. (**A**) Spectra on polyethylene highlighting the variability introduced by dyes. (**B**) Comparison between a selection of polyester spectra and polyethylene terephthalate spectra, which is a subclass of polyester. (**C**) Demonstrations of the important influence that laser wavelength selection has on resulting spectra, using PTFE, PE and PS as examples.

The inclusion of plastics with different colours and additives adds to the variety present in the database, because plastic with the same polymer structure but different colours can show significant spectral differences (Fig. 2a). The identity of plastic samples was taken from a sample’s source wherever possible, which includes all commercial samples and a subset of retail samples. Samples lacking direct identification, which includes some plastics originating from retail outlets and all samples originating from environmental sources, were manually assigned by comparison against standard spectra. Plastic samples assigned a source of *environmental* were donated by *Pollution Probe*, which is a not-for-profit organization. These samples were collected from the Great Lakes using patented Seabin technology^42^, predominantly from Lake Ontario and Lake Huron but specific location data is unavailable. The samples were cleaned prior to analysis by simply washing with de-ionized water and air-drying.

It should be noted that PET is a subtype of PEs. The assignments used here correspond to the supplier labels on each sample. Comparisons of spectra therefore show clear chemical differences between some PET and PEs samples, while others are very similar (Fig. 2b). Depending on research objectives, researchers may wish to merge PET and PEs into a single category.

### Raman spectra

Raman spectra were acquired using a Renishaw InVia Raman Microscope. Spectra were recorded between 100 and 4000 cm^−1^ using the SyncroScan measurement feature, which rocks the diffraction grating throughout the measurement to obtain a continuous spectrum of desired length. Spectra were acquired on samples using both a 633 nm laser and a 532 nm laser, using 1800 lines/mm and 2400 lines/mm diffraction gratings, respectively. The inclusion of two lasers intentionally introduces variations into a subset of the data, such as introduction of fluorescence processes (Fig. 2c). The laser-diffraction grating parings maintain comparably high spectroscopic resolution in the two spectra. Spectra on individual plastics are systematically identified using the plastic abbreviation, an ascending number denoting the unique samples within the plastic type, and ascending letters that denote multiple spectra of a single sample. Both the 532 nm and 633 nm lasers were calibrated by aligning the major peak from an internal silicon standard at 520.5 cm^−1^. All spectra are published in their raw (as-acquired) version. Processed spectra are also included in the RaSPI dataset to maximize compatibility and facilitate use. Processing of the RaSPI dataset involved (i) manual removal of cosmic rays facilitated by highlighting of potential cosmic rays using the feature width filtering capabilities in Renishaw WiRE software, (ii) interpolation and truncation to a 1 cm^−1^ grid between 225 and 3950 cm^−1^ to remove distortions at the edge of the spectra due to band-pass filters,(iii) algorithmic baseline subtraction using a the *improved modified polynomial* algorithm from the *pybaselines* package^43^, then (iv) intensity normalization between 0 and 1. The processing steps are shown in a Jupyter Notebook, *Raspi_Processing.ipynb*.

### Raman microscopic maps

The RaMPI dataset consists of 34 individual Raman microscopic maps. The maps were created by systematically acquiring Raman spectra across uniform 2-dimensional grids using a Renishaw InVia Raman Microscope. All spectra were acquired using a 633 nm laser with an 1800 line/mm diffraction grating. The diffraction grating was held static during measurements, with data recorded between 700 and 1800 cm^−1^. Experiments were performed using a 5X microscope objective that yields a laser spot diameter of 6.4 μm. The laser power, acquisition time per spectrum and accumulation time per spectrum were strategically varied across the different maps to introduce variations in noise, signal intensity and baseline.

The samples that were measured within the individual Raman microscopic maps consist of mixtures of plastics from the RaSPI dataset. Selected samples were ground to a micron-scale powder using metal graters, then cast onto a glass substrate. Spectroscopic maps acquired on samples from retail or commercial sources are named with a prefix “Map_”, while maps using environmentally sourced plastics are denoted as “Map_Env_”. Map names then contain a list of abbreviations for all plastics that exist within the spectroscopic map. For example, a map containing environmentally sourced POM, PP and PE will be named Map_Env_POM_PP_PE. The 34 maps contain a total of 33,119 spectra, where 16,519 correspond to spectra on plastics. The remaining 16,600 spectra are blank spectra on the space surrounding the particles.

### Spectroscopic Map classification

The RaMPI dataset includes arrays containing the assignment of every individual Raman spectrum within each map. These assignments were manually performed to ensure accuracy, with each individual spectrum assigned as one of the 14 labels used above, or as a ‘blank’ in the situation where the spectrum contains no plastic contributions. These labels are particularly useful for developing and testing ML models, especially considering the presence of multiple plastic samples and the variations in laser power and total acquisition across the different maps.

## Data Records

The RaSPI and RaMPI datasets are published as an open-source resource in the University of Waterloo DataVerse in Borealis^44^. This section is the primary source of information on the availability and content of the data being described. The root directory of the repository contains folders for each dataset.

The RaSPI folder includes comma separated value files containing the raw (RaSPI_raw.csv) and fully processed spectra (RaSPI_processed.csv). These files contain all RaSPI spectra alongside plastic identities. Metadata for each sample, which includes spectrum ID, colour, origin, additional info, plastic type, laser (nm) and diffraction grating (mm/l), is available in a separate file (RaSPI_metadata.csv). A Jupyter Notebook demonstrating the conversion of *RaSPI_raw.csv into RaSPI_processed.csv* is included (*RaSPI_processing.ipynb*).

The RaMPI folder contains folders for the spectroscopic maps and for the assignments of individual spectra. The spectroscopic maps are provided as raw spectra in the native Renishaw WiRE format to preserve the relevant measurement parameters, photographic images, and raw data. These files can be loaded through various methods, including an open source Python package, renishaw-wdf^41^. Assignments for individual spectra are provided in comma separated value files that include the manual plastic assignment and the intensity of the spectrum; all data was interpolated to a shared Raman shift axis, and the 2-dimensional data was unstacked to a 1-dimensional array. A Python pickle file is also included for convenience. This file contains a nested Python dictionary, with the Raman shift, raw intensity, photographic image array, and a 2 d array containing the assignments for each spectrum within the individual maps. Metadata pertaining to the RaMPI dataset is available in *RaMPI_metadata.csv*.

## Technical Validation

### Signal to noise ratio

**Fig. 3:**
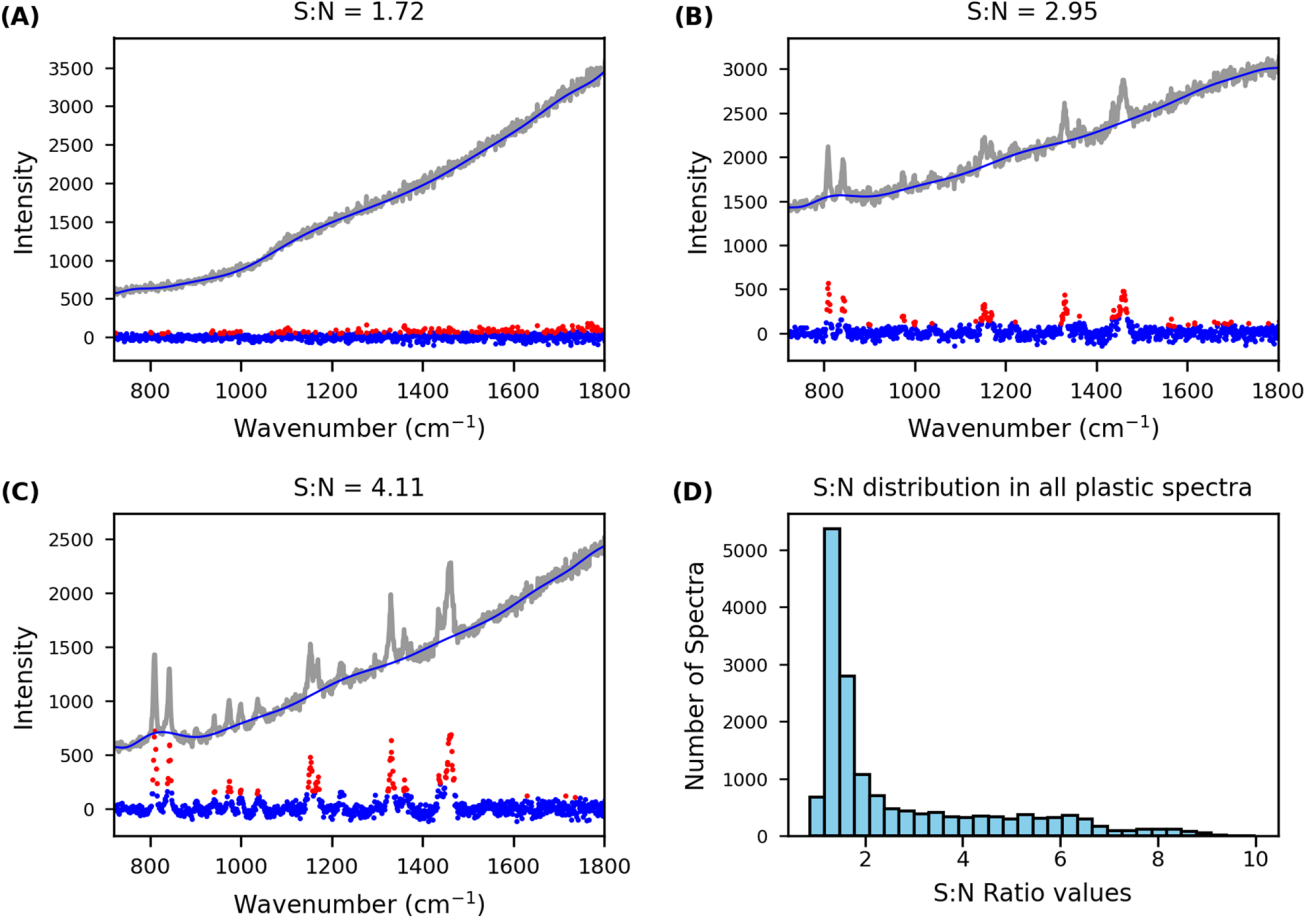
Application of algorithmic approach to inspect data for desirable variability in signal:noise ratios. Individual spectra from the map ‘Map_PP’ showing the algorithmic identification of “signal” from “noise” for (**A**) a blank spectrum, (**B**) a PP spectrum with mid-level signal:noise, and (**C**) a PP spectrum with a high-level signal:noise. (**D**) A histogram showing the signal:noise for all 16,519 plastic spectra across the 34 maps within RaMPI. Spectra shown include the raw and processed versions, with algorithmically determined baseline drawn and the signal and noise components identified.

Compared to the RaSPI dataset, the RaMPI dataset is intended to contain a larger number of spectra on a smaller number of samples. The dataset instead offers significant variations in the quality of spectra, which make the RaMPI dataset particularly useful as a test dataset for ML models. The SNR was calculated for all spectra to validate the variations. The SNR was calculated for all plastic-containing spectra in RaMPI by fitting a baseline, algorithmically distinguishing regions with signal from regions with only noise, then calculating the ratio of the regions. A baseline was fitted to individual spectra using the *improved modified polynomial baseline* (*imodpoly* function) algorithm in the *pybaselines* package^43^, which is an iterative polynomial fitting procedure that uses standard deviation of fit residuals to systematically remove data points until only baseline remains. This algorithm was used to separate the spectrum into signal and noise components, a 15^th^ order polynomial was fitted to the baseline component, then the baseline was subtracted from the total spectrum. This process yields noise as a random distribution of positive and negative values with balanced magnitudes, with spectroscopic signals spiking positive. The separated signal and noise subsets of data were used to calculate SNR ratios**:** signal was estimated as the mean of the 15^th^ to 5^th^ largest values from the signal component (chosen to avoid cosmic rays in a subset of spectra), while the 15^th^ to 5^th^ most negative values in the noise component provided an estimate of noise. Manual inspection confirms the accuracy of this algorithmic approach (Fig. 3). The 33,119 individual spectra contained within the 34 maps were first filtered to remove all 16,600 ‘blanks’ from consideration, leaving only the ‘plastic’ spectra. The algorithmic SNR was then calculated for all spectra within the dataset to confirm the desired variability across the data. The importance of these variations for plastics classification is shown in three spectra from the map ‘Map_PP‘, which simultaneously shows the algorithmic estimation of SNR and the variations in data quality between a ‘blank’ and two regions of polypropylene (Fig. 3a–c). The SNR for the remaining 16,519 spectra shows variation from 1–10 (Fig. 3d).

### Accuracy of interpolation

**Fig. 4:**
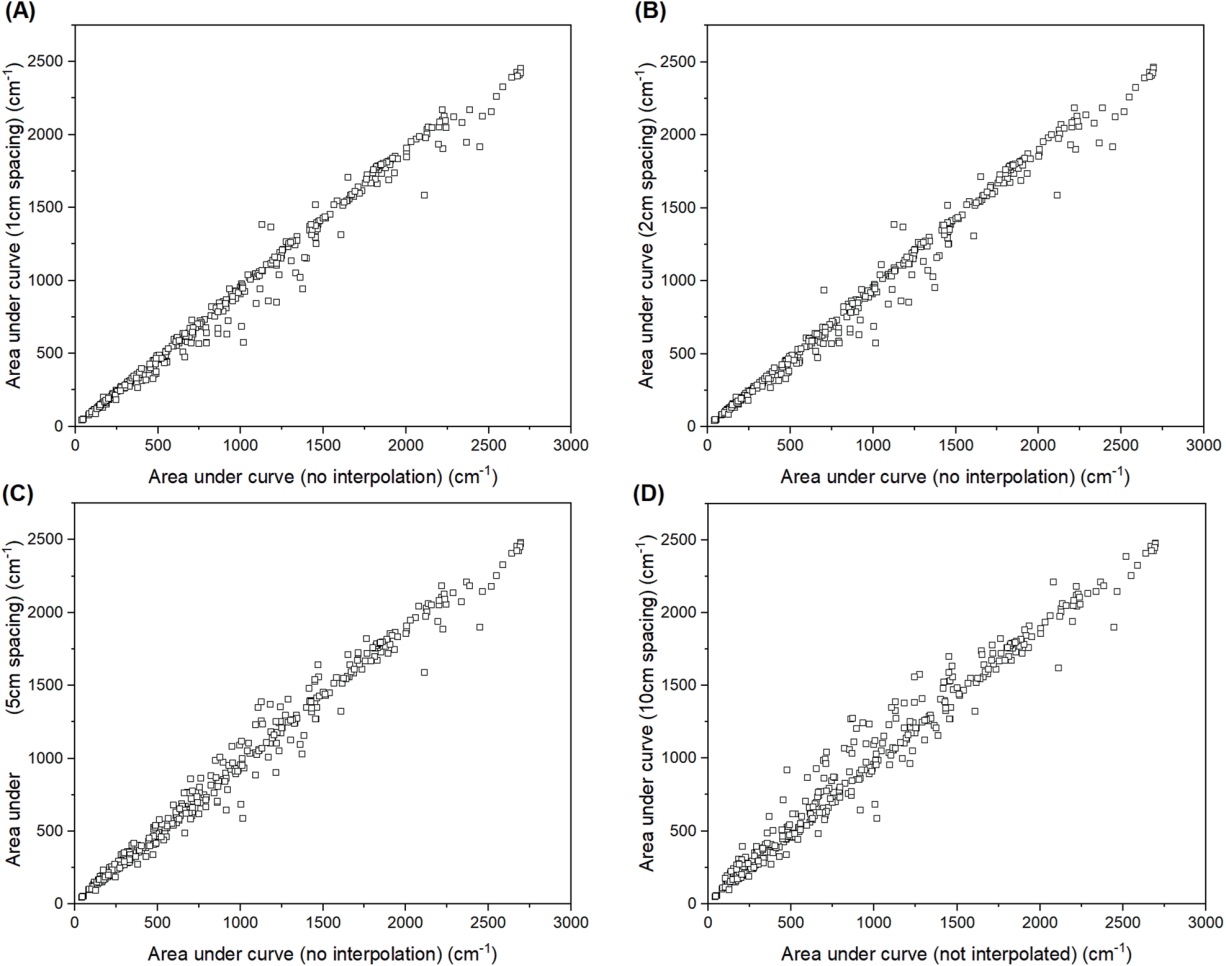
Correlations between the trapezoidal prism volume calculation for area beneath the curve of all spectra between non-interpolated spectra (x-axis A, B, C and D), and 1 cm (**A**), 2c m (**B**), 5 cm (**C**) and 10 cm (**D**) interpolated spectra.

The consistency of the interpolation process used in the RaSPI dataset was inspected by integration of the raw and processed spectra. The *trapz* function from the numpy^45^ package was used to integrate the non-interpolated data, and the spectra following interpolation at progressively higher wavenumber spacings (Fig. 4). The agreement between the integrated area with and without interpolation was inspected, with minimal deviation observed until *ca*. 10 cm^−1^ data spacing.

### Validation using ML models

**Fig. 5:**
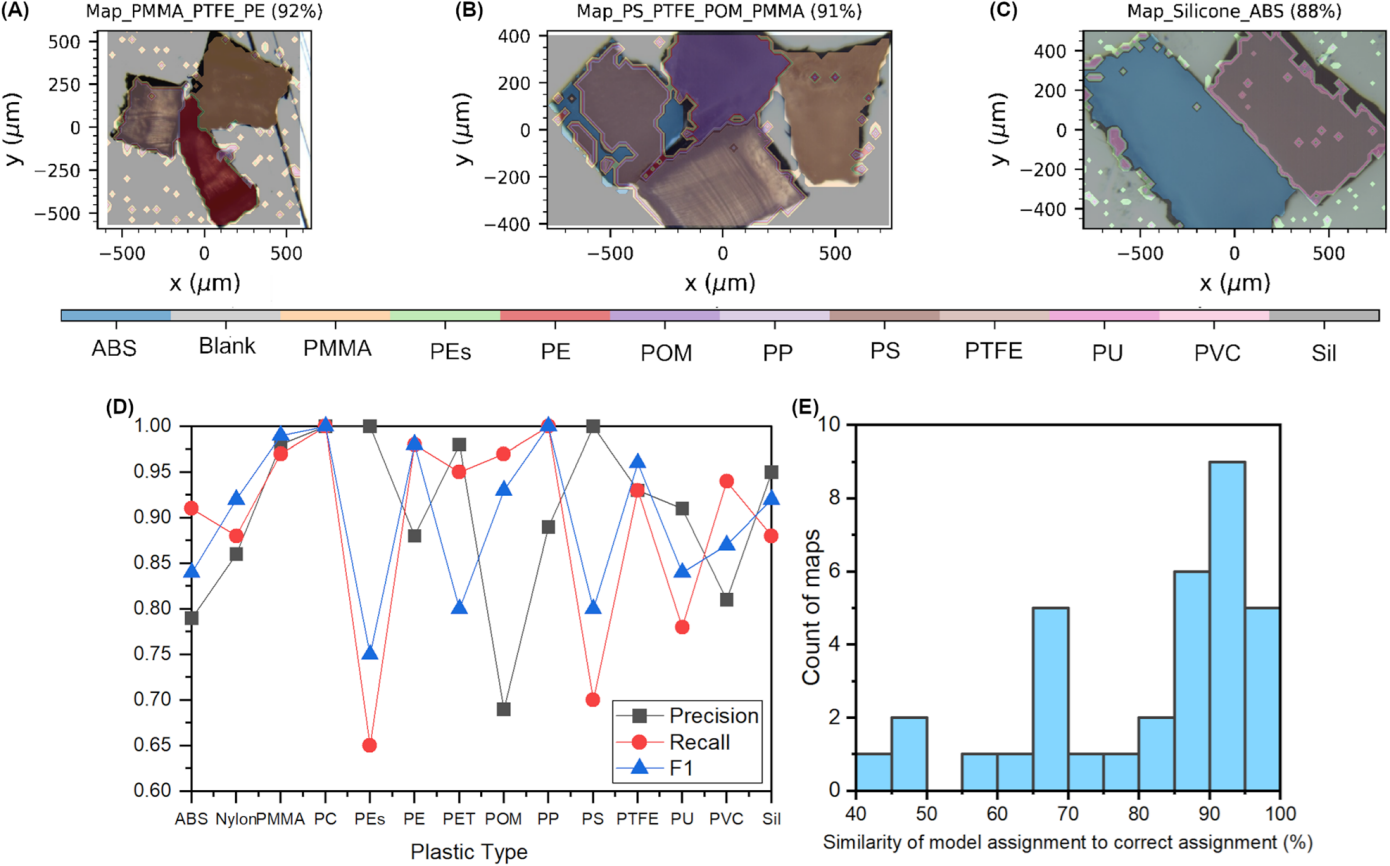
Results of a simple kNN model trained using the RaMPI dataset. (**A**–**C**) Assigned maps with % classification value and corresponding colour-key for plastics identification. (**D**) Precision, Recall and F1 values for each of the 14 plastics the model has been trained and tested on. (**E**) Histogram showing proportion of correct classifications across the 34 RaMPI maps.

The anticipated primary utility for RaMPI is for testing or validating ML model development. Three of the 34 maps in RaMPI have been previously used to test ML identification models trained on sections of the RaSPI dataset^31,34^. To further highlight the way that RaSPI and RaMPI can be used in conjunction with one another for plastics identification, a kNN model trained on the RaSPI dataset (processed version) was created using the ‘KNeighboursClassifier’ function provided by the scikit-learn python package^46^. This model was then tested on all 34 RaMPI maps, giving each spectra within these maps a classification as either ‘blank’, or one of the 14 plastic types. Excellent agreement can be seen between assignments from ML and those from manual assignment, shown by a selection of three of the RaMPI maps (Fig. 5a–c). The percentage classification value is shown next to the map names in (Fig. 5a–c) and in a histogram containing the % classification of all 34 RaMPI maps using this model (Fig. 5e). The precision, Recall and F1 values of this model for each of the 14 plastics it was trained on are also showcased (Fig. 5d). This comparison confirms the integrity of the RaMPI maps and compatibility between RaSPI and RaMPI, showcasing both datasets as useful and high-quality resources for training and testing of classification models for microplastics identification.

## Data Availability

Data is available at the University of Waterloo DataVerse in Borealis under CC-BY 4.0 license at 10.5683/SP3/8UQQQN.

## Code availability

Code associated with processing of the datasets is available alongside the dataset, under CC-BY 4.0 license at 10.5683/SP3/8UQQQN.

## References

[1] Baekeland, L. H. The Synthesis, Constitution, and Uses of Bakelite. *J. Ind. Eng. Chem.* **1**(3), 149–161, 10.1021/ie50003a004 (1909).

[2] Crespy, D., Bozonnet, M. & Meier, M. 100 Years of Bakelite, the Material of a 1000 Uses. *Angewandte Chemie International Edition* **47**(18), 3322–3328, 10.1002/anie.200704281 (2008). 18318037 10.1002/anie.200704281

[3] Ali Khan, M. U. *et al*. Bending Analysis of Polymer-Based Flexible Antennas for Wearable, General IoT Applications: A Review. *Polymers* **13**(3), 357, 10.3390/polym13030357 (2021). 33499265 10.3390/polym13030357 PMC7865813

[4] EA POD Report, https://plasticovershoot.earth/wp-content/uploads/2023/06/EA_POD_report_2023-V3.pdf (accessed 2024-09-13) (2023).

[5] *World Population Prospects 2022: Summary of Results | Population Division*. https://www.un.org/development/desa/pd/content/World-Population-Prospects-2022 (accessed 2024-09-13).

[6] Geyer, R., Jambeck, J. R. & Law, K. L. Production, Use, and Fate of All Plastics Ever Made. *Science Advances* **3**(7), e1700782, 10.1126/sciadv.1700782 (2017). 28776036 10.1126/sciadv.1700782 PMC5517107

[7] Van Cauwenberghe, L., Vanreusel, A., Mees, J. & Janssen, C. R. Microplastic Pollution in Deep-Sea Sediments. *Environmental Pollution* **182**, 495–499, 10.1016/j.envpol.2013.08.013 (2013). 24035457 10.1016/j.envpol.2013.08.013

[8] Padha, S., Kumar, R., Dhar, A. & Sharma, P. Microplastic Pollution in Mountain Terrains and Foothills: A Review on Source, Extraction, and Distribution of Microplastics in Remote Areas. *Environmental Research* **207**, 112232, 10.1016/j.envres.2021.112232 (2022). 34687754 10.1016/j.envres.2021.112232

[9] Race, T. O. New data reveals microplastics in world’s remotest ocean. *The Ocean Race*. https://archive.theoceanrace.com/en/news/11703_New-data-reveals-microplastics-in-worlds-remotest-ocean.html (accessed 2024-09-13).

[10] Smith, M., Love, D. C., Rochman, C. M. & Neff, R. A. Microplastics in Seafood and the Implications for Human Health. *Curr Envir Health Rpt* **5**(3), 375–386, 10.1007/s40572-018-0206-z (2018). 10.1007/s40572-018-0206-z PMC6132564 30116998

[11] Velebit, B., Janković, V., Milojević, L., Baltić, T. & Ćirić, J. Overview of Microplastics in the Meat: Occurrence, Detection Methods and Health Effects. *Scientific journal “Meat Technology”* **64**(2), 36–41, 10.18485/meattech.2023.64.2.6 (2023).

[12] Aydın, R. B., Yozukmaz, A., Şener, İ., Temiz, F. & Giannetto, D. Occurrence of Microplastics in Most Consumed Fruits and Vegetables from Turkey and Public Risk Assessment for Consumers. *Life* **13**(8), 1686, 10.3390/life13081686 (2023). 37629543 10.3390/life13081686 PMC10455475

[13] Prata, J. C., da Costa, J. P., Duarte, A. C. & Rocha-Santos, T. Methods for Sampling and Detection of Microplastics in Water and Sediment: A Critical Review. *TrAC Trends in Analytical Chemistry* **110**, 150–159, 10.1016/j.trac.2018.10.029 (2019).

[14] Amato-Lourenço, L. F. *et al*. Presence of Airborne Microplastics in Human Lung Tissue. *J Hazard Mater* **416**, 126124, 10.1016/j.jhazmat.2021.126124 (2021). 34492918 10.1016/j.jhazmat.2021.126124

[15] Leslie, H. A. *et al*. Discovery and Quantification of Plastic Particle Pollution in Human Blood. *Environment International* **163**, 107199, 10.1016/j.envint.2022.107199 (2022). 35367073 10.1016/j.envint.2022.107199

[16] Amato-Lourenço, L. F. *et al*. Microplastics in the Olfactory Bulb of the Human Brain. *JAMA Network Open* **7**(9), e2440018, 10.1001/jamanetworkopen.2024.40018 (2024). 39283733 10.1001/jamanetworkopen.2024.40018 PMC11406405

[17] De Tender, C. A. *et al*. Bacterial Community Profiling of Plastic Litter in the Belgian Part of the North Sea. *Environ. Sci. Technol.* **49**(16), 9629–9638, 10.1021/acs.est.5b01093 (2015). 26204244 10.1021/acs.est.5b01093

[18] Zhang, K. *et al*. Microplastic Pollution of Lakeshore Sediments from Remote Lakes in Tibet Plateau, China. *Environmental Pollution* **219**, 450–455, 10.1016/j.envpol.2016.05.048 (2016). 27238763 10.1016/j.envpol.2016.05.048

[19] Perraki, M. *et al*. Identification of Microplastics Using Μ-Raman Spectroscopy in Surface and Groundwater Bodies of SE Attica, Greece. *Water* **16**(6), 843, 10.3390/w16060843 (2024).

[20] Wiesheu, A. C., Anger, P. M., Baumann, T., Niessner, R. & Ivleva, N. P. Raman Microspectroscopic Analysis of Fibers in Beverages. *Anal. Methods* **8**(28), 5722–5725, 10.1039/C6AY01184E (2016).

[21] Dong, M. *et al*. Raman Spectra and Surface Changes of Microplastics Weathered under Natural Environments. *Science of The Total Environment* **739**, 139990, 10.1016/j.scitotenv.2020.139990 (2020). 32535468 10.1016/j.scitotenv.2020.139990

[22] Wiesinger, H., Wang, Z. & Hellweg, S. Deep Dive into Plastic Monomers, Additives, and Processing Aids. *Environ. Sci. Technol.* **55**(13), 9339–9351, 10.1021/acs.est.1c00976 (2021). 34154322 10.1021/acs.est.1c00976

[23] Hahladakis, J. N., Velis, C. A., Weber, R., Iacovidou, E. & Purnell, P. An Overview of Chemical Additives Present in Plastics: Migration, Release, Fate and Environmental Impact during Their Use, Disposal and Recycling. *Journal of Hazardous Materials* **344**, 179–199, 10.1016/j.jhazmat.2017.10.014 (2018). 29035713 10.1016/j.jhazmat.2017.10.014

[24] Andrady, A. L. *et al*. Oxidation and Fragmentation of Plastics in a Changing Environment; from UV-Radiation to Biological Degradation. *Science of The Total Environment* **851**, 158022, 10.1016/j.scitotenv.2022.158022 (2022). 35970458 10.1016/j.scitotenv.2022.158022 PMC9765214

[25] Bao, R. *et al*. Secondary Microplastics Formation and Colonized Microorganisms on the Surface of Conventional and Degradable Plastic Granules during Long-Term UV Aging in Various Environmental Media. *Journal of Hazardous Materials* **439**, 129686, 10.1016/j.jhazmat.2022.129686 (2022). 36104912 10.1016/j.jhazmat.2022.129686

[26] Chamas, A. *et al*. Degradation Rates of Plastics in the Environment. *ACS Sustainable Chem. Eng.* **8**(9), 3494–3511, 10.1021/acssuschemeng.9b06635 (2020).

[27] Cruz, J. N., Martínez, K. D., Zavariz, Á. D. & Hernández, I. P. Review of the Thermochemical Degradation of PET: An Alternative Method of Recycling. *Journal of Ecological Engineering*, **23**(9), 10.12911/22998993/151766 (2022).

[28] Doğan, M. Ultraviolet Light Accelerates the Degradation of Polyethylene Plastics. *Microscopy Research and Technique* **84**(11), 2774–2783, 10.1002/jemt.23838 (2021). 34046978 10.1002/jemt.23838

[29] Cai, L., Wang, J., Peng, J., Wu, Z. & Tan, X. Observation of the Degradation of Three Types of Plastic Pellets Exposed to UV Irradiation in Three Different Environments. *Science of The Total Environment*, **628–629**, 740–747, 10.1016/j.scitotenv.2018.02.079 (2018). 10.1016/j.scitotenv.2018.02.079 29454214

[30] Weber, F., Zinnen, A. & Kerpen, J. Development of a Machine Learning-Based Method for the Analysis of Microplastics in Environmental Samples Using µ-Raman Spectroscopy. *Microplastics and Nanoplastics* **3**(1), 9, 10.1186/s43591-023-00057-3 (2023).

[31] Lei, B. *et al*. Customizable Machine-Learning Models for Rapid Microplastic Identification Using Raman Microscopy. *Anal. Chem.* **94**(49), 17011–17019, 10.1021/acs.analchem.2c02451 (2022). 36445839 10.1021/acs.analchem.2c02451

[32] Gonçalves, G., Andriolo, U., Gonçalves, L., Sobral, P. & Bessa, F. Quantifying Marine Macro Litter Abundance on a Sandy Beach Using Unmanned Aerial Systems and Object-Oriented Machine Learning Methods. *Remote Sensing* **12**(16), 2599, 10.3390/rs12162599 (2020).

[33] Feng, Z., Zheng, L. & Liu, J. Classification of Household Microplastics Using a Multi-Model Approach Based on Raman Spectroscopy. *Chemosphere* **325**, 138312, 10.1016/j.chemosphere.2023.138312 (2023). 36907487 10.1016/j.chemosphere.2023.138312

[34] Hogan, Ú. E., Voss, H. B., Lei, B. & Smith, R. D. L. Integrating C–H Information to Improve Machine Learning Classification Models for Microplastic Identification from Raman Spectra. *Anal. Chem.* **97**(4), 2214–2222, 10.1021/acs.analchem.4c05197 (2025). 39818746 10.1021/acs.analchem.4c05197

[35] Cabernard, L., Roscher, L., Lorenz, C., Gerdts, G. & Primpke, S. Comparison of Raman and Fourier Transform Infrared Spectroscopy for the Quantification of Microplastics in the Aquatic Environment. *Environ. Sci. Technol.* **52**(22), 13279–13288, 10.1021/acs.est.8b03438 (2018). 30350953 10.1021/acs.est.8b03438

[36] Marica, I. & Pînzaru, S. C. A Raman Spectral Database of Naturally Aged Plastics: A Proof-of-Concept Study for Waste Plastic Sorting. *Journal of Raman Spectroscopy* **54**(3), 305–313, 10.1002/jrs.6484 (2023).

[37] Miller, E. A. *et al*. A Raman Spectral Reference Library of Potential Anthropogenic and Biological Ocean Polymers. *Sci Data* **9**(1), 780, 10.1038/s41597-022-01883-5 (2022). 36566263 10.1038/s41597-022-01883-5 PMC9790010

[38] Munno, K., De Frond, H., O’Donnell, B. & Rochman, C. M. Increasing the Accessibility for Characterizing Microplastics: Introducing New Application-Based and Spectral Libraries of Plastic Particles (SLoPP and SLoPP-E). *Anal. Chem.* **92**(3), 2443–2451, 10.1021/acs.analchem.9b03626 (2020). 31939281 10.1021/acs.analchem.9b03626

[39] Cowger, W. *et al*. Microplastic Spectral Classification Needs an Open Source Community: Open Specy to the Rescue! *Anal. Chem.* **93**(21), 7543–7548, 10.1021/acs.analchem.1c00123 (2021). 34009953 10.1021/acs.analchem.1c00123

[40] Cowger, W. *et al*. Critical Review of Processing and Classification Techniques for Images and Spectra in Microplastic Research. *Appl Spectrosc* **74**(9), 989–1010, 10.1177/0003702820929064 (2020). 32500727 10.1177/0003702820929064

[41] Henderson, A. Renishaw File Reader, 10.5281/zenodo.495477 (2017).

[42] *Nature Certificates: Combatting Microplastics and Ocean Pollution*. https://seabin.io/home (accessed 2024-05-14).

[43] Erb, D. Pybaselines: A Python Library of Algorithms for the Baseline Correction of Experimental Data. **2024**. 10.5281/zenodo.5608581 (2024).

[44] Smith, R. D. L., Hogan, U. E., Voss, H. B., Bec, A. E. & Feng, X. Replication Data for: Raman Spectra for Plastics Identification (RaSPI) and Raman Maps for Plastics Identification (RaMPI) Research. *Borealis*10.5683/SP3/8UQQQN (2025). 10.1038/s41597-026-07103-8 PMC13194819 41912531

[45] Harris, C. R. *et al*. Array Programming with NumPy. *Nature* **585**(7825), 357–362, 10.1038/s41586-020-2649-2 (2020). 32939066 10.1038/s41586-020-2649-2 PMC7759461

[46] Pedregosa, F. *et al*. Scikit-Learn: Machine Learning in Python. *Journal of Machine Learning Research* **12**(85), 2825–2830 (2011).

